# Headache Secondary to Isolated Sphenoid Sinus Fungus Ball: Retrospective Analysis of 6 Cases First Diagnosed in the Neurology Department

**DOI:** 10.3389/fneur.2018.00745

**Published:** 2018-09-07

**Authors:** Xiaoyu Gao, Bing Li, Maowen Ba, Weidong Yao, Chunjuan Sun, Xuwen Sun

**Affiliations:** ^1^Department of Neurology, The Affiliated Yantai Yuhuangding Hospital of Qingdao University, Yantai, China; ^2^Department of Pathology, The Affiliated Yantai Yuhuangding Hospital of Qingdao University, Yantai, China; ^3^Department of Radiology, The Affiliated Yantai Yuhuangding Hospital of Qingdao University, Yantai, China

**Keywords:** isolated sphenoid sinus fungus ball (SSFB), headache, endoscopic transnasal sphenoidotomy, diagnosis, clinical feature

## Abstract

Fungal sphenoid sinusitis is easily misdiagnosed in clinic, particularly for patients with normal immunological status. Due to the anatomic characteristics of sphenoid sinus, patients presented with various nonspecific symptoms and complications. Headache is the most common presentation, but location of headache is not fixed. We intended to analyze 6 cases of headache secondary to the isolated sphenoid sinus fungus ball (SSFB) which were first diagnosed in the Neurology Department. There was significant female predominance with mean ages of 55 years. They had repeatedly headache history from months to years. The headache was unilateral and usually on the side of lesions. Medication of pain relievers worked well in the beginning of SSFB, but not in the late stage of disease. Notably, all patients did not present positive nervous systemic signs. A preoperative computed tomography (CT) scan or magnetic resonance imaging (MRI) demonstrated the inflammation in sphenoid sinus. Some cases showed calcification in soft tissue or bone lesions of sinus wall. All of 6 patients undertook transnasal endoscopic sphenoidotomy without antifungal therapy after operation. Characteristic fungus ball (FB) was detected after histopathological examination. No headache recurrence was found after average 15.5 months follow-up. Our results suggested that transnasal endoscopic sphenoidotomy is the treatment of choice to remove the FB in sphenoid sinus with a low rate of morbidity and recurrence.

## Introduction

Fungal rhinosinusitis can be broadly classified as invasive and noninvasive fungal infection according to histopathology. The pathological features of noninvasive fungal rhinosinusitis are fungal infection confined to the paranasal sinuses, mucous membrane, and bone wall without fungal invasion, including fungus ball (FB) and allergic fungal rhinosinusitis (1, 2). FB is a noninvasive dense accumulation of fungal hyphae in one sinus cavity, usually the maxillary sinus (1, 2). The sphenoid sinus is the second most frequent site of this disease. The traditional opinion that fungal sinusitis mainly occurs in long-term use of antibiotics, oral steroids, immunosuppressive agents, radiation therapy, and some chronic wasting diseases (such as diabetes, large area burns) patients (3). But, FB has been reported with increasing frequency over the last two decades. Isolated sphenoid sinus fungus ball (SSFB) are easily misdiagnosed due to the nonspecific symptoms and undetectable anatomical location. Headache is the most common symptom presented by patients (4). The sphenoid sinus lies in close proximity to important anatomic structures, including the cavernous sinus, the optic nerve, the internal carotid artery, and cranial nerves III, IV, V, and VI. Thus, spread of infection or inflammation beyond the sphenoid sinus to these neighboring structures may result in serious intracranial and orbital complications. Although the inflammation of FB mainly localizes in the sinus cavity without lesions of mucus and bone wall, it can lead to absorbent thinning of sinus wall. It has been reported that 63% of FB will cause lesions of bone and further damage the organs in proximity (5). Fungal sphenoid sinusitis is not often found in the clinic, particularly for patients with normal immunological function and without the history of taking oral steroids (3). Therefore, most FB cannot be diagnosed for the first time. Paranasal sinus CT scan is a useful exam for the diagnosis of fungal sphenoid sinusitis (5–7).

In the present study, we sought to retrospectively analyze on how effectively diagnose and treat of 6 sphenoid sinus fungus ball patients in our institution.

## Methods

Approval from Institutional Review Board and Ethics Committee of Qingdao University for retrospective studies was obtained before commencing the study. The study followed the tenets of the Declaration of Helsinki. Six patients were collected from September 2013 through September 2017 in the affiliated Yantai Yuhuangding Hospital of Qingdao University. Their ages ranged from 46 to 59 years. All patients had normal immunologic status. No abnormal nervous systemic signs were found by neurological examinations. Further diagnosis of sphenoid sinus lesions were found in cranial CT or MRI. Then, these patients were referred to the Otolaryngology Department. Paranasal sinus CT hinted fungal sphenoid sinusitis. All of the 6 patients were treated by transnasal endoscopic sphenoidotomy without antifungal therapy, and histopathology diagnosis was isolated sphenoid sinus fungus ball (SSFB). Prognosis was determined by phone calls and no headache recurrence occurred during follow-up.

## Results

### Case 1

**Figure 1:**
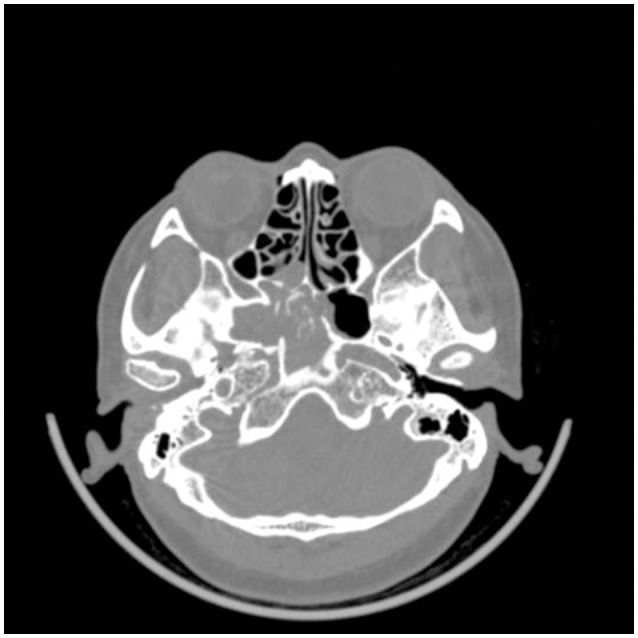
CT image of paranasal sinus. Irregular soft tissue intensity was observed in right sphenoid sinus with high density of point stripe calcification and lesion on the sinus wall.

**Figure 2:**
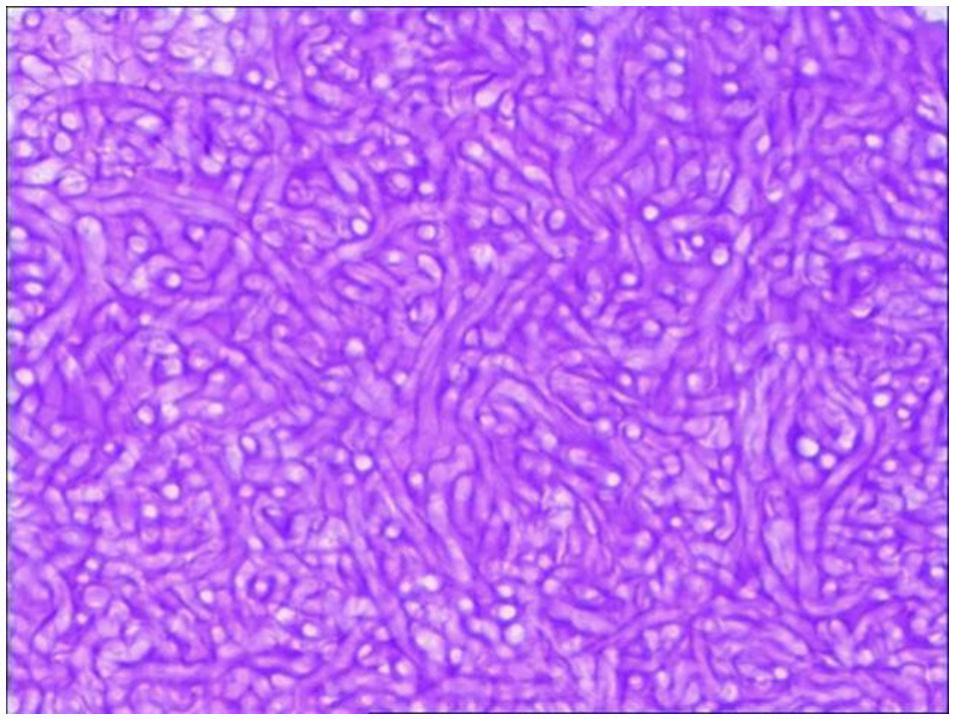
Pathological diagnosis of fungus ball. The operation tissue was cut into slides with H&E stain. The vesicular structure was identified as aspergillus fumigatus through light field microscope (40 × amplification).

A 48-year-old woman visited out-patient clinic of neurology after 2 weeks of headache. The localization of headache was mainly on right side of head. It showed as intermittent dull pain, especially at night. Headache was relieved after taking pain reliever. However, headache occurred again when she stopped taking medicine. The patient visited out-patient clinic of neurology again. There was no abnormal change in brain parenchyma after taking brain CT scan. However, occupying lesion was found in the right sphenoid sinus. This patient was immediately hospitalized in the Otolaryngology Department. Further paranasal sinus CT demonstrated abnormal density of the right sphenoid sinus with calcification and lesion on the sinus wall (Figure 1). Patient was treated by transnasal endoscopic sphenoidotomy without antifungal therapy. Histopathological examination diagnosed right sphenoid sinus as SSFB (Figure 2). No further recurrence of her headache was found after 14 months follow-up.

### Case 2

A 46-year-old woman visited out-patient clinic of neurology after 1 month of headache. The localization of headache was mainly on left forehead. It showed as persistent dull pain, especially at night. Patient had occasional dizziness. After taking medicine prescribed from community hospital, headache relieved, but could not cure. Then, patient visited our out-patient clinic of neurology. There was no positive symptoms in nervous system. Brain MRI exam demonstrated that few demyelination in white matter of both frontal lobe and inflammation in sphenoid sinus. This patient was then hospitalized in the Otolaryngology Department. Further paranasal sinus CT demonstrated inflammation in left sphenoid sinus. Patient undertook transnasal endoscopic sphenoidotomy without antifungal therapy. Histopathological examination diagnosed left sphenoid sinus as SSFB. No further recurrence of her headache happened after 11 months follow-up.

### Case 3

A 66-year-old woman was admitted in the Neurology Department because of 2 month repeatedly headache, aggravating within 1 week. The localization of headache was mainly on left side of forehead, tempus, and cheek. It showed as intermittent pain, worse in the morning and tended to aggravate when she lowered her head. 1 week before she was admitted in hospital, headache was persistent accompanied by upper teeth pain on left side. She had hypertension (blood pressure 177/88 mmHg) and occasional nasal bleeding. There was no positive symptoms in nervous system. Brain CT imagine showed scattered demyelination in white matter of both frontoparietal lobes and inflammation in the left maxillary and sphenoid sinus. Further enhanced Brain MRI also demonstrated scattered demyelination in white matter of both frontoparietal lobes, inflammation of paranasal sinuses, and mucoceles in the left sphenoid sinus. Then, this patient was referred to the Otolaryngology Department. Further paranasal sinus CT demonstrated abnormal intensity of soft tissue in left sphenoid sinus, protrusion to the sinus cavity with nodular calcification. Patient was treated by transnasal endoscopic sphenoidotomy without antifungal therapy. Histopathological examination diagnosed left sphenoid sinus as SSFB. Follow-up of 10 months demonstrated no further recurrence of her headache.

### Case 4

A 61-year-old woman visited out-patient clinic of neurology because of 1 year intermittent distending pain on the right occipital, aggravating within 10 days. She received treatment from the community hospital but without relief. There was no positive symptoms in nervous system. CT scan did not show any abnormal alteration in brain parenchyma. It indicated right fungal sphenoid sinusitis. Then, this patient was referred to the Otolaryngology Department. Paranasal sinus CT abnormal intensity of soft tissue in right sphenoid sinus with calcification, bone damage on sinus wall, fungal sphenoid sinusitis. Patient undertook transnasal endoscopic sphenoidotomy without antifungal therapy. Histopathological examination diagnosed right sphenoid sinus as SSFB. Follow-up of 5 months demonstrated no further recurrence of her headache.

### Case 5

A 50-year-old woman visited out-patient clinic of neurology because of 1 year intermittent sharp pain on the left head and face, aggravating within 15 days. In the past 1 year, headache occurred every day and lasted for 3–4 h, symptom was worse at day time, particularly after bending. There was no nausea and vomiting. Gradually, pain localized on left cheek, accompanying with left superior teeth and eye pain. She was treated with Vitamin B1, Mecobalamin and Carbamazepine, but without relief. There was clear tenderness on the position of left supraorbital nerve and infraorbital nerve. She was preliminarily diagnosed as trigeminal neuralgia and was admitted in the Neurology Department. Enhanced brain MRI showed left sphenoid sinusitis with mucoceles, but without any abnormal alteration in brain parenchyma. Consultation of ENT doctor suggested to perform paranasal sinus CT scan. It demonstrated that left sphenoid sinusitis with abnormal intensity of soft tissue, absorbent thinning of front sinus wall, bone hyperplasia of side and rear sinus wall. Then, this patient was referred to the Otolaryngology Department. Patient was treated by transnasal endoscopic sphenoidotomy without antifungal therapy. Histopathological examination diagnosed left sphenoid sinus as SSFB. No further recurrence of her headache was found after 4 years follow-up.

### Case 6

A 59-year-old woman visited out-patient clinic of neurology because of 3 years intermittent sharp pain on left orbital. She was diagnosed as trigeminal neuralgia in the community hospital. Then, she was treated with Carbamazepine, Mecobalamin and acupuncture, but without significant relief. Our examination found tenderness at the exit of the left supraorbital nerve. MRI imagine demonstrated that partial empty sella and left sphenoid sinus. This patient was referred to the Otolaryngology Department. Paranasal sinus CT demonstrated inflammation of left sphenoid sinus. Patient was treated by transnasal endoscopic sphenoidotomy without antifungal therapy. Histopathological examination diagnosed left sphenoid sinus as SSFB. Follow-up of 2 month demonstrated no further recurrence of her headache.

All of the major clinical information in patients with SSFB was summarized in the Table 1.

**Table 1:** Clinical characteristics of 6 patients.

**Case number**	**Age**	**Gender**	**Headache**			**Brain CT**	**Brain MRI**	**Paranasal sinus CT**	**Operation**	**Histopathology**
			**Durations**	**Position**	**Characteristics**					
1	48	Female	2 w	Right occipital	Intermittent dull pain,tends to be worse in the evening	Space-occupying lesions in the right sphenoid sinus	No	Irregular soft tissue intensity was observed in right sphenoid sinus with high density of point stripe calcification and lesion on the sinus wall.	Transnasal endoscopic sphenoidotomy	FB of right sphenoid sinus
2	46	Female	1 m	Left frontal	Persistent dull pain, and tends to be worse in the evening	No	Demyelination in white matter of both frontal lobe and inflammation in left sphenoid sinus	Inflammation of left sphenoid sinus	Transnasal endoscopic sphenoidotomy	FB of left sphenoid sinus
3	66	Female	2 m	Left fronto-temporal,cheek and superior alveolar bone	Intermittent distending pain,aggravated by bending	Scattered demyelination in white matter of both frontoparietal lobes, inflammation in the left maxillary and sphenoid sinus	Scattered demyelination in white matter of both frontoparietal lobes, inflammation of paranasal sinuses, and mucosal cysts in the left sphenoid sinus	Abnormal intensity of soft tissue in left sphenoid sinus, protrusion to the sinus cavity with nodular calcification	Transnasal endoscopic sphenoidotomy	FB of left sphenoid sinus, chronic inflammation of mucous tissue
4	61	Female	1 y	Right occipital	Intermittent distending pain	Inflammation in right sphenoid sinus	No	Abnormal intensity of soft tissue in right sphenoid sinus with calcification,bone damage on sinus wall, fungal sphenoid sinusitis	Transnasal endoscopic sphenoidotomy	FB of right sphenoid sinus
5	50	Female	1 y	Left fronto-temporal, cheek, and superior alveolar bone	Intermittent sharp pain, and aggravated by bending	No	Left sphenoid sinusitis	Abnormal intensity of soft tissue in left sphenoid sinus, absorbent thinning of front sinus wall, bone hyperplasia of side and rear sinus wall	Transnasal endoscopic sphenoidotomy	FB of left sphenoid sinus, chronic inflammation of mucous tissue
6	59	Female	3 y	Left orbital	Intermittent sharp pain, aggravate gradually and interferes with sleep at last	No	Left sphenoid sinusitis	Inflammation of left sphenoid sinus	Transnasal endoscopic sphenoidotomy	FB of left sphenoid sinus

## Characteristics of headaches

There was no etiological factors to cause headache in all 6 cases. We summarized these characteristics of headaches in the Table 2. Briefly, the onset of headache was usually intermittent and lasted for couple of minutes or hours per day in the beginning of SSFB. The frequency of headache gradually increased as time was prolonged. Medication of pain relievers worked well in the early stage of SSFB, but not in the late stage of disease. Additionally, headache of SSFB was unilateral and usually on the lesion side. The headache was aggravated by coughing, lowering head, or exertion in some cases. Importantly, all patients did not have abnormal nervous systemic signs.

**Table 2:** Characteristics of headache in fungal sphenoid sinusitis.

**Case**	**Duration of attacks**	**Pain location**	**Headache associated symptoms**	**Relationship of headache with possible precipitating factors**	**Headache severity score and effects on the headaches after work or family activities**	**Acute and preventive medications tried in the past**	**Presence of co-existing conditions that may influence treatment of choice**
1	Continuous	Right occipital	No	Tends to be worse in the evening	4, without aggravation after work	Paracetamol	No
2	Continuous	Left frontal	No	Tends to be worse in the evening,	6, without aggravation after work	Ibuprofen	No
3	3–4 h every morning, continuous in recent 3 weeks	Left fronto-temporal, cheek and superior alveolar bone	Occasional nasal bleeding	Aggravated by lowering head	5, without aggravation after work	Ibuprofen	Hypertension
4	1–2 h every day, continuous in recent 10 days	Right occipital	No	exertion	3, without aggravation after work	Mecobalamin,Loxoprofen	No
5	Couple of minutes, 3–4 times per day, continuous in recent 1 month	Left fronto-temporal, cheek and superior alveolar bone	No	Lower head and cough	5, in the beginning, gradual aggravation; 8, when visited out-patient clinic	Vitamin B1. Mecobalamin, Carbamazepine	No
6	Ten minutes per time, one time per day or per couple of days,frequency gradually increased, continuous in recent 1 month	Left orbital	Intermittent nasal discharge	Aggravated by cough and exertion	3, in the beginning without aggravation after daily work; being worse to 7 one month before visiting out-patient clinic	Acupuncture, Vitamin B1,Mecobalamin,Carbamazepine	No

## Treatment and outcome

All surgical procedures were performed under general anesthesia (100%). Six patients all undertook transnasal endoscopic sphenoidotomy surgery. All patients exhibited clay-like material that indicated of FB. Then, histopathological examination confirmed aspergilloma in all patients.

Total healing was usually obtained between 3 and 6 weeks after operation. All patients were followed up at least for 2 months with a mean of 15.5 months. All cases with headache reported complete resolution after surgery.

## Discussion

SSFB is a rare disease in clinic, particularly for patients with normal immunological status. Unlike invasive forms, a SSFB is a noninvasive form of fungal growth in the sinuses (8). However, growing studies including ours demonstrated that SSFB occurs in immunocompetent hosts (9–11). The pathogenesis of paranasal sinus FB is unclear. Due to the nonspecific symptoms of SSFB (12), it is very easy to be misdiagnosed in clinic. Furthermore, the important anatomic location of sphenoid sinus, SSFB patients need to be treated at early stage, avoiding further lesions or spreading to proximity structures (13). Our results suggested that SSFB should be considered in patients with unexplained headache, especially in elderly women. Imaging techniques such as CT or MRI are effective ways to exclude the further intracranial lesions and give useful clues concerning inflammation in sinus.

More evidence has indicated that the case number of SSFB is increasing over the last two decades (9, 10). One major reason is attributed to the widely using of imaging techniques, including CT and MRI, to find causes of unexplained headache. In addition to detect the lesions in sinus, brain CT or MRI scans are very useful for the clinicians to differential diagnosis of SSFB (9–11, 14). Additionally, aging of the population might be another factor to have more SSFB in clinic (9, 10). Our 6 cases were all female patients with average ages of 55 years old. In line with our results, compelling groups have reported that SSFB mainly occurs in elderly patients with a female predominance (15–20). Notably, bacterial infection may influence the development and persistence of clinical symptoms in a substantial portion of fungus ball cases (21). Most SSFB patients have the antibiotics treatment history before they are diagnosed with SSFB. The abuse of broad-spectrum antibiotics, which may result in the imbalance of the bacterial flora in human body and create an environment to promote fungal growth (9, 10, 21). Also, the relationship between intranasal anatomy and maxillary FB is thought to play an important role, but with different conclusions. Hwang et al. observed that the middle meatus bears the major part of the inspiratory nasal airflow, and the relatively larger volume of the middle meatus was associated with the localization of the FB (22). In contrast, Tsai et al. (23) reported that the anatomical variants do not predispose patients to paranasal sinus FB. Additionally, dentogenic factors may correlate with the presence of maxillary sinus fungus ball (24). One proposed theory is dental filling material containing zinc oxide which may promote maxillary fungal growth (25). In our 6 SSFB patients, we did not observe the clear anatomic variations and teeth implants.

In summary, headaches are the most commonly presented in SSFB patients (6, 26). Our six patients all have the history of headache with various location and different symptoms and complications but without positive nervous systemic signs. The pain relievers works well in the beginning of SSFB, but not in the late stage of disease. All of these characteristics are helpful for the clinicians to differential diagnosis of headache secondary to the SSFB. Transnasal endoscopic sphenoidotomy is considered as a treatment of choice to remove FB completely and reestablish proper ventilation and drainage in sphenoid sinus (27). All in all, our study provided an important rationale for the clinician to effectively diagnose and treat the SSFB patients with good prognosis and frequent resolution of headaches.

## Conclusion

SSFB should be suspected in patients with unexplained unilateral headache. Paranasal sinus CT scan is a useful technique for the quick diagnosis of SSFB. Transnasal endoscopic sphenoidotomy is an effective therapy to remove the FB in sphenoid sinus with a low rate of morbidity and recurrence.

## Ethics statement

The descriptive study was approved by ethical committee of the Affiliated Yantai Yuhuangding Hospital of Qingdao University. All participants provided informed consent for academic publication.

## Author contributions

XG and XS contributed to the conception and design of the research, prepared for the initial manuscript draft. XG, BL, and MB collected the clinical data and literature research. WY contributed to the pathological diagnosis. CS contributed to the imaging diagnosis. All authors have read and approved the final manuscript.

### Conflict of interest statement

The authors declare that the research was conducted in the absence of any commercial or financial relationships that could be construed as a potential conflict of interest.
